# Studies of Angular Resolution for Acoustic Arc Arrays

**DOI:** 10.3390/s23136007

**Published:** 2023-06-28

**Authors:** Dmitry A. Sednev, Alexey I. Soldatov, Andrey A. Soldatov, Maria A. Kostina, Dmitry O. Dolmatov, Daria A. Koneva

**Affiliations:** School of Non-Destructive Testing, National Research Tomsk Polytechnic University, 30 Lenin Avenue, 634050 Tomsk, Russia

**Keywords:** ultrasonic nondestructive testing, phased arrays, acoustic arc arrays, concave arrays, convex arrays, flexible phased arrays, total focusing method, angular resolution, Rayleigh criterion

## Abstract

Currently, phased arrays are increasingly used in ultrasonic nondestructive testing. One of the most important parameters of ultrasonic nondestructive testing with the application of phased arrays is the angular resolution. This paper presents the results of studies of the angular resolution of concave and convex acoustic arrays in ultrasonic testing with the application of the total focusing method. Computer modeling of concave and convex acoustic arrays consisting of 16, 32 and 64 elements with distances between elements of 0.5 and 1 mm and arc radii of 30 and 60 mm have been performed. The results obtained by computer modeling were confirmed via in situ experiments.

## 1. Introduction

Ultrasonic testing is a non-replaceable tool for many industrial applications of non-destructive testing. Methods of ultrasonic testing are used for the inspection of welded joints, pressure vessels, pipelines, forgings, rolled sheets and other products. Multi-element acoustic arrays have been used in non-destructive testing since the end of the 20th century. The phased array ultrasonic testing technique was implemented to manipulate the acoustic beam, as it does not require many computational tools [1]. With computers emerging there appeared the possibility to realize complex computations, providing complex algorithms to manipulate multi-element acoustic arrays. The most widespread method is the total focusing method (TFM), named the “Golden Standard”, as well as its modifications [2,3,4,5,6,7,8,9,10,11,12]. In the majority of industries such as aviation, aerospace and nuclear, the ultrasonic testing of products is very often performed with direct contact or with the application of a prism which is located between the acoustic array and the object under inspection. During inspection of an object with a curvilinear surface, the fixed form of an array and prism create a non-regular interlayer which leads to a change in the beam trajectory and reduces the inspection efficiency.

One of the approaches implemented to address this limitation may be joining a flat array with a prism which has a special form appropriate for the surface of the object under inspection [13]. However, in this case an individual prism is required for each surface; this becomes impractical for many applications as the surface profile of an object under inspection changes. A more common approach involves joining a flat acoustic array with a flexible prism [14] or with a water-immersed camera or flexible membrane [15,16,17,18,19]. In these cases, first, the object surface profile is identified; then, the required delay laws are calculated for each transducer–receiver couple using the Fermat principle [20,21] for the restoration of the inner structure of an object under inspection using the TFM. The effect of a product having a non-regular profile (independent of whether the probe is used during contact or immersion) on the inspection result was investigated by the authors S. Mahaut, J. Porre, P. Calmon, S. Chatillion and O. Roy [22]. The authors discovered that during the emission of ultrasonic waves through an anisotropic structure, there is observed a small change of the refraction angle as well as a deeper focusing point, in the case that the same delay law is applied to the probe as during the irradiation through isotropic media. When the transducer is placed above an irregular profile, beam distortion is observed, as the delay law is calculated in relation to the flat interface boundary; the focusing may occur in another location, or the beam may not be focused.

The application of flexible ultrasonic arrays may be the solution to the problem in the case that the possibility to adapt regular ultrasonic arrays to products with complex geometry is absent [23,24,25,26,27,28,29,30]. Several companies have launched production of such arrays; among these companies are those such as Olympus, Doppler, Imasonic and others. During restoration of the image of the inspection zone tomogram using the TFM, it is necessary to know the coordinates of the flexible array elements; the array is located on the curvilinear surface of the object under inspection. The solution to this problem can be found in the interconnection of the flexible acoustic array with a fiber-optical sensor which is mounted above the array and measures its curvature. Based on these data, the coordinates of the acoustic array elements are calculated [31]. With this design, the probe’s overall size is not large, which is convenient for multiple applications. Furthermore, no prior knowledge about the product geometry is needed; this makes this approach a universal one. To determine the radius of curvature for the fiber-optical cable, intensity reduction of the optical radiation passing through the fiber-optical cable is used. However, limitations related to the fiber-optical sensor disallow the inspection of products with a curvature radius of less than 125 mm.

Studies of the acoustic field of a probe with a curved array were performed in previous research [32]. A probe with a curved matrix contained a number of elements uniformly distributed on the arc. For non-decaying homogeneous and isotropic media with the longitudinal velocity *c* and with the density *ρ*, the acoustic pressure at a point in the field may be calculated with the Rayleigh–Sommerfeld integral:

(1) $$p=\frac{j\rho c}{\lambda }\sum_{i=1}^{N}\frac{exp(jk\xi )}{\xi }dS_{n},$$

where *j* is the imaginary unit, λ is the wavelength, *S_n_* is the surface area of the *n*-numbered element, ξ is the distance from the point transducer on the *n*-numbered element to the point in the field and *N* is the number of elements in the array.

For experimental studies of the acoustic beam profile in compliance with the ASTM E 1065-99 standard [33], the authors used a reflector in the shape of a ball made from stainless steel with a diameter of 6 mm. The probes were submerged in water and positioned above the reflector.

The calculations performed by the authors for the probe with an arc array demonstrated that the distribution of axial acoustic pressure represents a single peak, and its amplitude monotonously increases with the increase of focus depth infinitely. The real focus positions were different from the set values and approached the center of the probe circumference. For the probe with an arc array, the irregularity of its acoustic field was caused by the coexistence of electrical and natural focusing, which affected the distribution of the acoustic field in the inspection zone [34].

The conducted analysis revealed that flexible acoustic arrays are finding increasing applications in non-destructive inspection of products with curvilinear surfaces. The category of such products is wide: pipelines, crankshafts of combustion engines, bearings, airstream propellers of gas turbine engines, lock bars, etc. In addition, multichannel flaw detectors are most widely used with 16, 32 or 64 channels; they are able to process the data from acoustic arrays with the same number of elements. The range of product diameters may vary from 10 mm for bearings used for measuring equipment up to 200 mm for bearings used in transport machines for railroads, automobiles and aeronautics. The crankshaft for a combustion engine is the most complicated, in terms of manufacturing, and highly costly product with a curved surface. High operational loads determine a heightened level of requirements for the quality of crankshaft manufacturing. Different techniques of destructive as well as non-destructive inspection are applied to identify defects in crankshafts [35,36,37,38,39,40]. During the inspection of such products, it is necessary to know the resolution of the inspection system.

However, no research has been conducted to determine the resolutions of flexible acoustic arrays. Due to this, it seems to be a relevant goal to study the dependence of the angular resolution on different parameters of ultrasonic testing with the TFM’s application: the curvature of the phased array, the number of elements in it and the distance between elements in the probe.

**Figure 1:**
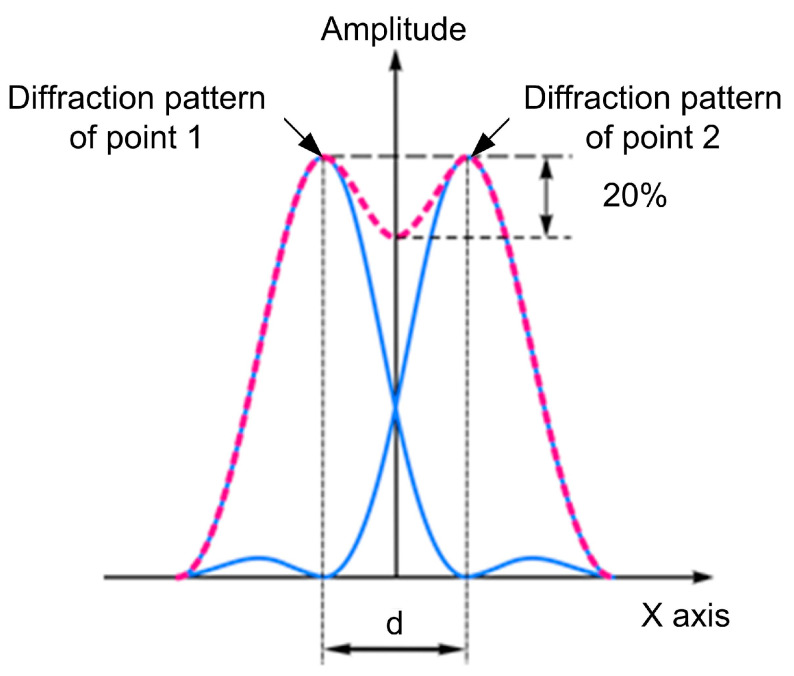
Resolution according to the Rayleigh criterion.

The resolution is one of the main parameters of any inspection technique. It characterizes the minimum linear and angular distance between two defects positioned at the same distance and which are differentiated by the flaw detector and registered separately from one another in accordance with the Rayleigh criterion. The Rayleigh criterion for the diffraction limit of resolution states that two images are just resolvable when the center of the diffraction pattern of one is directly over the first minimum of the diffraction pattern of the other. At the same time, the decrease of the amplitude between the two maximums should be no less than 20% (Figure 1).

## 2. Modeling

**Figure 2:**
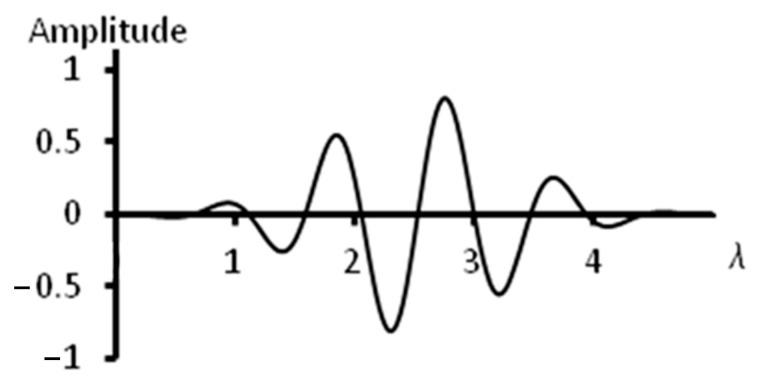
The waveform of an echo-signal.

**Figure 3:**
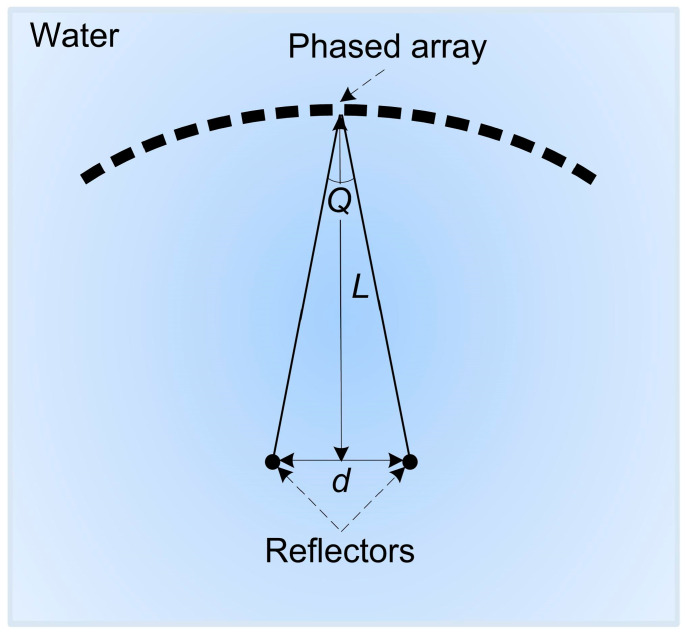
Scheme of modeling.

The studies of the resolutions of curved arrays were conducted using computer modeling based on the TFM’s principles [34]. In modeling, arrays of convex and concave configurations were used with curvature radii of 30 and 60 mm and with numbers of elements of 16, 32 and 64, as these are the values most widely used in non-destructive inspection. The chosen range of curvatures corresponds to the sizes of crankshaft main-bearing journals and crankshaft connecting-rod journals of trucks. For example, for a KAMAZ truck, the radius of the crankshaft main-bearing journals is 47 mm, while the radius of the crankshaft connecting-rod journals is 40 mm. In a SCANIA truck, those values are 69.5 mm and 52 mm, respectively. In addition, there is wide range of nomenclature for bearings of the same sizes. In accordance with these sizes, a range of reflector locations of 5, 10, 15, 25, 35 and 45 mm was selected. The wavelength was 0.3 mm. The chosen wavelength is conditioned by the minimum size of the detected defects. In the modeling software are set the coordinates of two reflector locations; then, for each radiation source–receiver pair, an A-scan is generated. The A-scan contains echo-signals from each reflector. The type of echo-signal used in the modeling is given in Figure 2. Point defects were used as reflectors in the performed computer modeling. These defects were placed symmetrically relative to the acoustic axis of the phased array. The scheme of the modeling performed is shown in Figure 3. Letter L in the figure indicates the depth of the reflectors’ locations, and d shows the distance between reflectors.

The echo-signals were located on the time axis of the A-scans in relation to the time delay equal to the time of elastic wave propagation in the pathway of radiation source–reflector–receiver. The obtained set of A-scans is then used for restoration of the inspection zone using the TFM, which involves the calculation of the amplitude for each pixel of the inspection zone by summing up the amplitudes of all A-scans with the time delay.

**Figure 4:**
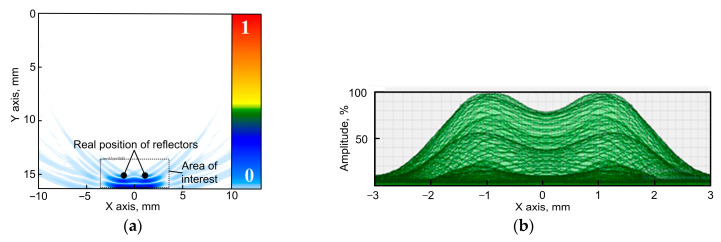
Modeling result (an array of 16 elements with an arc radius of 60 mm and distance between the elements of 1 mm; reflectors are located at a depth of 15 mm; the distance between reflectors is 0.55 mm). (**a**) Image of the flaws obtained using TFM; (**b**) distribution of the amplitudes of the two reflectors.

Figure 4a demonstrates the result of the restoration of the image of the inspection zone with two reflectors. The reflectors are located at a depth of 15 mm, and the distance between reflectors is 0.55 mm. A concave phased array of 16 elements with an arc radius of 60 mm and a distance between elements of 1 mm was applied in that case. The acoustic image of the reflectors is given in blue color at the bottom of Figure 4a. The real positions of the reflectors are represented by two circles of black color. The modeling software allows the presentation of the obtained amplitudes of pixels in the set inspection zone. The zone dimensions are chosen by the user. A Hilbert transform is performed for this zone, while the obtained amplitude distribution for this zone is presented in the dependence diagram. It is possible to present the absolute value of the amplitude as well as the relative value, which is determined by each pixel amplitude’s division by the maximum amplitude in this zone, which is convenient for calculations of the resolution according to the Rayleigh criterion. Figure 4b demonstrates the relative distribution of amplitudes of the two reflectors after performing a Hilbert transform for the zone of the two reflectors’ locations as it is given in Figure 4a. The maximum value of the amplitude and the difference between the two maximums are determined based on the obtained distribution of the amplitudes of the two reflectors. This allows for the evaluation of the resolution of the inspection system.

**Figure 5:**
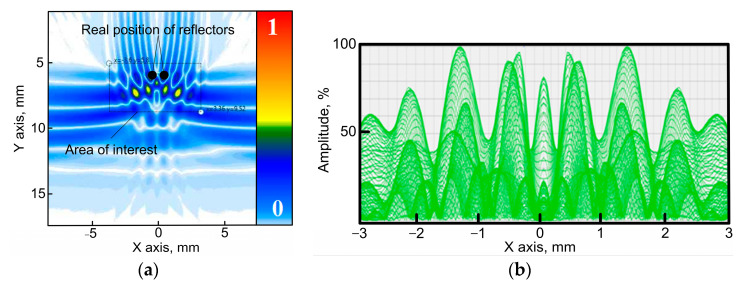
Modeling result (an array of 16 elements with an arc radius of 60 mm and distance between elements of 1 mm; the reflectors are located at a depth of 6 mm; the distance between reflectors is 0.3 mm). (**a**) Image of the flaws obtained using TFM; (**b**) distribution of the amplitudes of the two reflectors.

The modeling results demonstrated that at distances of 10 mm and less, the side lobes appear and do not allow the detection of defects (Figure 5). Figure 5a provides the image of the inspection zone, while Figure 5b demonstrates the distribution of the amplitudes of two reflectors located at a depth of 6 mm; the distance between reflectors is 0.3 mm. The phased array applied in this case consists of 16 elements, with a distance between elements of 1 mm and an arc radius of 60 mm.

The distance between reflectors and their depth were varied during the modeling. The simulation results were used to estimate the angular resolution. The results for a fixed configuration of a concave acoustic array and fixed depth of flaws were considered as one case. For each case, the minimal distance between the reflectors at which the Rayleigh criterion was met was determined. Fulfillment of the Rayleigh criterion means that flaws could be resolved as a result of TFM processing. The obtained value was used to evaluate the angular resolution of the acoustic array using the following equation:

(2) $$Q=2\cdot \arctan\frac{d_{\min}}{2L},$$

where *d*_min_ is the minimal distance between the reflectors at which the Rayleigh criterion was met.

**Figure 6:**
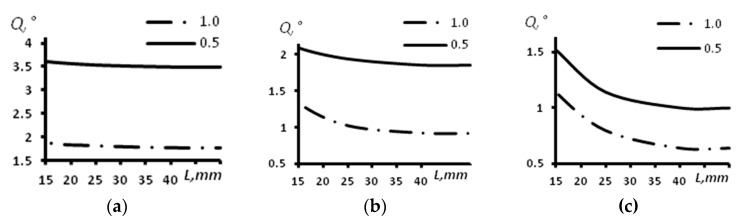
The dependence of the angular resolution on the distance for different sizes of the aperture of the concave array with a radius of 60 mm. (**a**) Acoustic array of 16 elements; (**b**) Acoustic array of 32 elements; (**c**) Acoustic array of 64 elements.

The results of angular resolution estimation for a concave arc array with a radius of 60 mm using computer modeling are presented in Table 1 and Figure 6. The dependence of the angular resolution (*Q*) on the distance (*L*) for different sizes of the aperture of the array of 16 elements is given in Figure 6a. The dependence of the angular resolution on the distance for different sizes of the aperture of the array of 32 elements is presented in Figure 6b. The dependence of the angular resolution on the distance for different sizes of the aperture of the array of 64 elements is given in Figure 6c. The continuous line corresponds to a distance between the array elements of 0.5 mm, and the dash–dot line corresponds to a distance between the elements of the array of 1.0 mm.

From the analysis of Table 1 and Figure 6, it is possible to conclude that the angular resolution is enhanced with the increase of the array aperture. At a distance greater than 30 mm the resolution almost does not change, while it slightly increases at distances from 15 mm to 30 mm. Particularly large enhancements occurred for the arrays with 64 elements and aperture sizes of 64 and 32 mm. The change comprised about 0.5 degrees. For the array of 16 elements with an aperture of 16 mm, the angular resolution changed from 1.90 degrees at a distance of 15 mm to 1.78 degrees at a distance of 45 mm. The decrease of the aperture by half down to 8 mm led to a change in the angular resolution from 3.61 degrees at a distance of 15 mm to 3.49 degrees at a distance of 45 mm. For the array of 32 elements with an aperture of 32 mm, the angular resolution changed from 1.33 degrees at a distance of 15 mm to 0.92 degrees at a distance of 45 mm. The decrease of the aperture by half down to 16 mm led to a change in the angular resolution from 2.09 degrees at a distance of 15 mm to 1.85 degrees at a distance of 45 mm.

**Figure 7:**
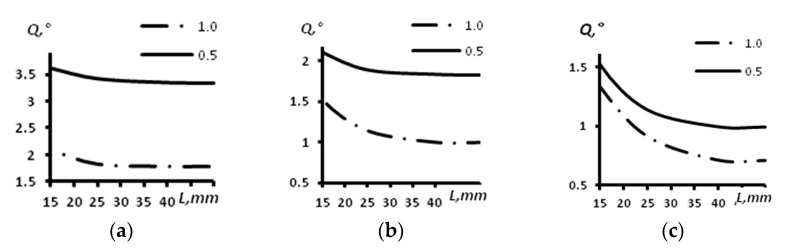
The dependence of the angular resolution on the distance for different sizes of the aperture of a concave array with radius of 30 mm. (**a**) Acoustic array of 16 elements; (**b**) Acoustic array of 32 elements; (**c**) Acoustic array of 64 elements.

The change of the curvature radius of the concave array up to 30 mm led to a decrease in angular views, which led to a resolution decrease. The obtained results are presented in Table 2 and Figure 7. The dependence between the angular resolution and the depth of the reflectors’ location for the probe with 16 elements is presented in Figure 7a. The same results for the arrays with 32 and 64 elements are presented in Figure 7b,c, respectively. In all the aforementioned figures, a continuous line indicates a distance between the elements of the array of 0.5 mm, and the dash–dot line indicates a distance between the elements of the array equal to 1.0 mm.

The obtained results demonstrate that in comparison with the concave arc array with a curvature radius of 60 mm, for the concave array with a curvature radius of 30 mm the angular resolution is worse in most of the cases. For the array of 16 elements with an aperture of 16 mm, the angular resolution for a distance of 15 mm was 2.09 degrees, whereas this value for the same probe with an arc radius of 60 mm was 1.90 degrees. For the concave array of 16 elements with an aperture of 8 mm and an arc radius 30 mm, the angular resolution at a depth of 15 mm was the same as it was for the concave probe with a curvature radius of 60 mm. For the probe with 32 elements and an aperture size of 32 mm, the angular resolution values were 1.52 and 1.33 degrees at a depth of 15 mm and arc radii of 30 and 60 mm, correspondingly. For the concave phased array with 64 elements and an aperture of 64 mm, the angular resolution at a depth of 15 mm was 1.33 degrees (for the probe with an arc radius of 30 mm) and 1.14 degrees (for the probe with an arc radius of 60 mm).

At distances greater than 30 mm, the angular resolution did not change and was 1.78, 1.00 and 0.71 degrees for the arrays of 16, 32 and 64 elements and with a distance between elements of 1 mm, respectively. For the same arrays with a distance between elements of 0.5 mm, it was 3.34, 1.83 and 1.00 degrees, respectively.

**Figure 8:**
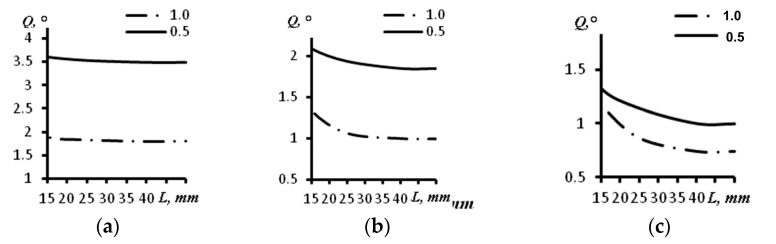
The dependence of angular resolution on the distance for different sizes of the aperture of the convex array with radius of 60 mm. (**a**) Acoustic array of 16 elements; (**b**) Acoustic array of 32 elements; (**c**) Acoustic array of 64 elements.

The modeling performed for the convex arrays revealed a similar character of dependence of the angular resolution on the distance. Figure 8 demonstrates the dependences of the angular resolution on the distance for the convex arrays with a curvature radius of 60 mm, with different numbers of elements and different aperture sizes.

The results obtained for angular resolution are presented in Table 3. Dependencies between the angular resolution and the distance of the reflectors’ locations for the phased arrays with 16, 32 and 64 elements are presented in Figure 8a–c. In these figures, a continuous line indicates a distance between the elements of the array of 0.5 mm, and a dash–dot line represents a distance between the elements of the array equal to 1.0 mm.

Comparing Figure 6 and Figure 8, it is possible to make the conclusion that the angular resolution for the convex and concave arrays with a curvature radius of 60 mm is the same practically at all the distances. The most significant difference between the convex and concave phased arrays was obtained for the probes with 64 elements and a distance between elements of 0.5 mm; it was equal to 0.19 degrees.

**Figure 9:**
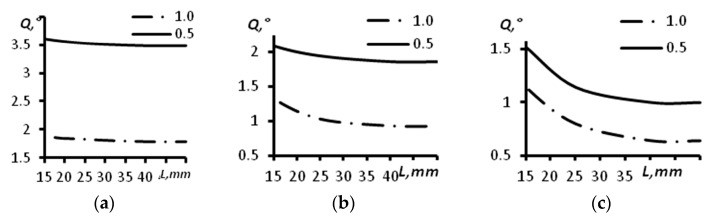
The dependence of the angular resolution on the distance for different sizes of the aperture of the convex array with a radius of 30 mm. (**a**) Acoustic array of 16 elements; (**b**) Acoustic array of 32 elements; (**c**) Acoustic array of 64 elements.

The values of the angular resolution obtained for the convex array of 30 mm and various depths of reflector location are presented in Table 4. Figure 9 demonstrates the dependences of the angular resolution on the distance for convex arrays with a curvature radius of 30 mm, different numbers of elements and different aperture sizes. The dependence of the angular resolution on the distance for different sizes of aperture of the array of 16 elements is presented in Figure 9a. The dependence of the angular resolution on the distance for different sizes of aperture of the array of 32 elements is given in Figure 9b. The dependence of the angular resolution on the distance for different sizes of aperture of the array of 64 elements is provided in Figure 9c. Continuous lines correspond to a distance between the elements of the array of 0.5 mm, and dash–dot lines correspond to a distance between the elements of the array of 1.0 mm.

A comparison of Figure 7 and Figure 9 leads to the conclusion that the angular resolution for the convex and concave arrays with a curvature radius of 30 mm slightly differs. Thus, for the array of 16 elements with an aperture of 16 mm, a difference is observed only for the distance of 15 mm (0.19 degrees). At other distances, the resolutions of the two considered shapes of the phased array are similar. For the same array with an aperture of 8 mm, the resolution is the same for all the distances. For the array of 32 elements and an aperture of 32 mm, a difference is observed only at a distance of 15 mm (0.19 degrees), while with an aperture of 16 mm, the difference was 0.06 degrees (the distance is 15 mm) and decreased to 0 at a distance of 35 mm. For the array of 64 elements with an aperture of 64 mm, the change amounted to 0.38 degrees (the distance is 15 mm) and decreased to 0 at a distance of 35 mm, while for the array with an aperture of 32 mm, the difference amounted to 0.19 degrees (the distance is 15 mm) and decreased to 0 degrees at a distance of 25 mm.

**Figure 10:**
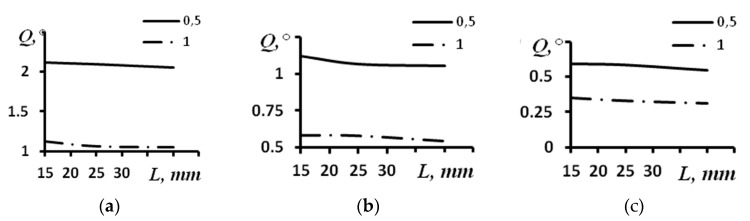
The dependence of the angular resolution on the distance for different sizes of the aperture of the array with infinite curvature. (**a**) Acoustic array of 16 elements; (**b**) Acoustic array of 32 elements; (**c**) Acoustic array of 64 elements.

Studies using the models of similar arrays with infinite curvature radii were conducted for comparison. The modeling results for the arrays with different parameters are given in Table 5 and Figure 10. The dependence of the angular resolution on the distance for different sizes of the aperture of the array of 16 elements is presented in Figure 10a. The dependence of the angular resolution on the distance for different sizes of the aperture of the array of 32 elements is given in Figure 10b. The dependence of the angular resolution on the distance for different sizes of the aperture of the array of 64 elements is presented in Figure 10c. Continuous lines correspond to a distance between the array elements of 0.5 mm, and dash–dot lines correspond to a distance between the array elements of 1.0 mm.

From the analysis of Table 5 and Figure 10, it is possible to conclude that the angular resolution is enhanced in direct proportion to enhancement of the aperture. For the array of 16 elements, the aperture’s enhancement by 2 times led to the increase of the resolution also by about 2 times from 2.06 degrees to 1.05 degrees. Similarly, for the arrays with 32 and 64 elements, the changes were from 1.05 degrees to 0.54 degrees and from 0.54 degrees to 0.31 degrees, correspondingly.

## 3. The Experimental Studies

**Figure 11:**
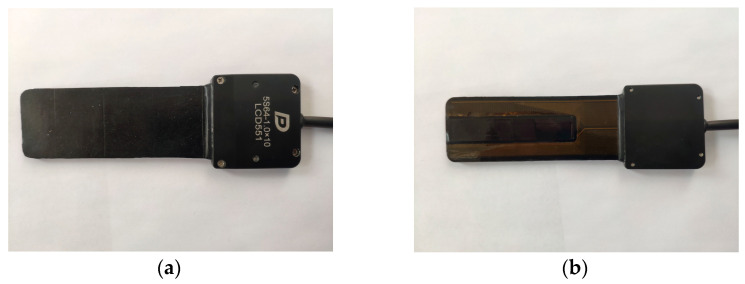
Doppler 5S64-1.0×10 multi-element flexible acoustic array. (**a**) Top view; (**b**) Bottom view.

**Figure 12:**
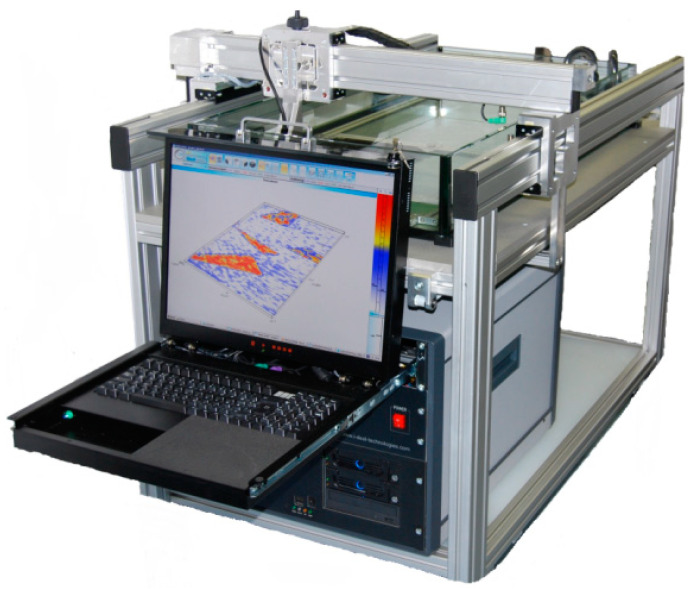
Multichannel ultrasonic inspection system: Idealsystem.

To verify the results obtained using computer simulation, a series of in situ experiments was carried out. A DOPPLER 5S64-1.0×10 flexible phased array was used as a probe in the experiments (Figure 11). The probe consists of 64 elements with a pitch of 1 mm and a central frequency of 5 MHz. The IDEALSYSTEM multichannel ultrasonic testing system (IDEAL-Technologies GmbH) was used to control the elements of the probe (Figure 12). Due to the parameters of the ultrasonic control system, only 16 elements of the flexible phased array were active during the experiments and taking in emitted ultrasonic waves and receiving echo-signals.

**Figure 13:**
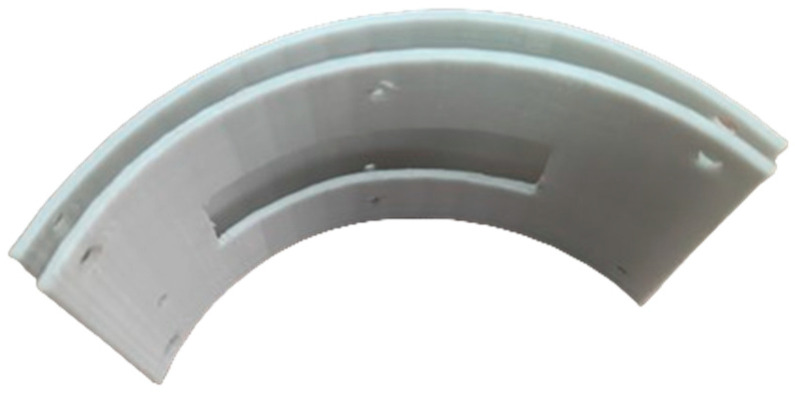
The special mold for the flexible phased array.

**Figure 14:**
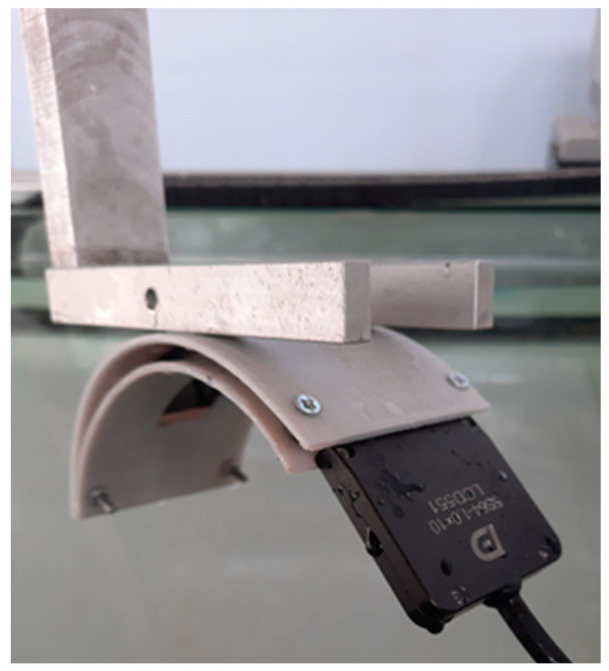
The flexible phased array being set up in the molds.

For an experimental study of the angular resolution, a special mold was made using 3D printing (Figure 13). This mold provided the concave shape of a flexible phased array with a radius of 60 mm. The application of curvature of such a radius is conditioned by the technical capacities of the applied probe. According to the technical requirements, the curvature radius of the array 5S64-1.0×10 comprises 60 mm. Thus, a concave probe with 16 elements with an aperture size of 16 mm and a curvature radius of 60 mm was considered in experiments. The options for positioning the flexible phased array on the experimental set-up are given in Figure 14.

Two reflectors with diameters of 0.5 mm were used in the experiments. The reflectors were manufactured using 3D printing. Polylactic acid was used as the material for 3D printing. The flaws were placed on a thread, which was attached to a U-shaped plate. The reflectors were placed under water during the experiments. Thus, the wavelength of longitudinal ultrasonic waves was 0.3 mm during experimental verification.

During the study of the angular resolution, the reflectors were sequentially placed at distances of 10, 15, 20, 25 and 45 mm relative to the central element of the array. The study technique comprised five stages. The first stage presupposes the subsequent emission of elastic waves produced by each element of the array and reception of the reflected waves by all elements of the flexible phased array. During the second stage the restoration of the inspection zone is performed with the TFM. At the third stage the zone with the reflectors is chosen on the obtained tomogram. A Hilbert transform is performed for this zone at the fourth stage, and the dependence diagram for the amplitude distribution in this zone is restored. At the fifth stage, the amplitude is measured in the center of the diffraction image for each reflector and among them. Based on the measurement results, the resolution is estimated in accordance with Equation (2).

**Figure 15:**
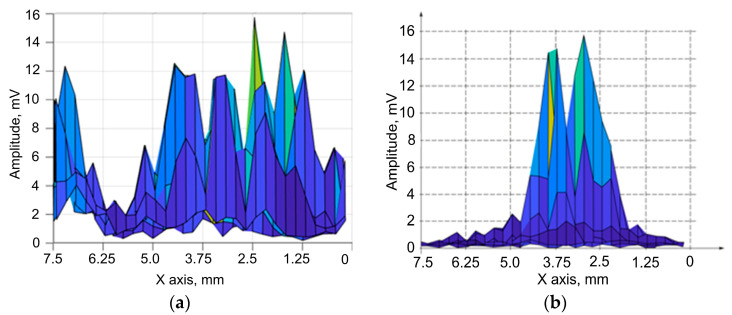
The amplitude distribution in the zone of the two reflectors’ locations. (**a**) A distance of 10 mm; (**b**) A distance of 20 mm.

Figure 15 demonstrates the amplitude distribution dependences in the zone of the two reflectors’ locations. Figure 15a shows the amplitude distribution dependences of the two reflectors located at a distance of 10 mm from the central element of the concave flexible acoustic array. The amplitude distribution obtained for the concave probe with the reflector located at a distance of 20 mm is presented in Figure 15b.

As can be seen from Figure 15a, multiple peaks are observed at a distance of 10 mm. This is related to the presence of the side lobes of the flexible acoustic arc array. At this distance it is not possible to detect two defects. At a distance of 20 mm, two defects are detected separately (Figure 15b). The values of the angular resolution obtained for different distances of the reflectors’ locations are shown in Table 6.

For distances of 20, 25, 35 and 45 mm, a good correlation between the experiment and the modeling was obtained. The relative error for these cases did not exceed 9%. The significant difference for a depth of 15 mm (relative error of 21%) is due to different conditions of the modeling and in situ experiments. In the modeling, each element of the curved probe was considered as a point, whereas in the utilized flexible phased array, the size of the element was 0.8 × 10 mm. This caused a difference in the beam patterns of the probe’s element between the experiment and in the modeling. Therefore, during the in situ experiments, echo-signals from the reflectors at low distances were not received by the edge elements of the array, whereas this took place in the modeling.

## 4. Conclusions

As a result of the studies of the angular resolutions for different configurations of transducers with 16, 32 and 64 elements, with distances between elements of 0.5 mm and 1 mm and arc radii of 30 and 60 mm, it was observed that it was not possible to detect separately two defects at distances of up to 15 mm for the considered arrays when the wavelength was 0.3 mm. The character of the dependence of the angular resolution on the distance is similar for all the studied arrays. The worst resolution is observed at a distance of 15 mm, and with the increase of the distance it enhances up to a distance of 30 mm, while at distances higher than 30 mm it does not change. For the arrays of 16 and 32 elements, the enhancement is no less than 10%, while for the array of 64 elements, the enhancement is significantly higher and ranges from 20% to 50%.

At distances greater than 30 mm, the angular resolution of the arrays approximates the resolution of the linear acoustic arrays with an aperture equal to the shortest distance between the edge elements of the arc array. Thus, the aperture size produces the main effect on the angular resolution of the arc arrays. The greater the aperture is in size, the better the angular resolution is. The convex and concave arrays with the same aperture size possess the same approximate angular resolution at distances greater than 30 mm.

These studies have shown that during testing of products with curved surfaces (crankshafts and camshafts of internal combustion engines, blades of gas turbine engines, blades of water and steam turbines, structural elements of bearings (rollers, rings), wheels and axles of railway cars, etc.) changes in the angular resolution of the curved phased array should be considered in both of the following cases: when changing the curvature radius of the object and when changing the depth at which inspection is performed.

## Figures and Tables

**Table 1:** Result of angular resolution evaluation for the concave probe with a radius of 60 mm.

Depth. mm	Number of Elements in the Probe					
	16		32		64	
	Distance between Elements. mm					
	1	0.5	1	0.5	1	0.5
15	1.90	3.61	1.33	2.09	1.14	1.52
25	1.82	3.53	1.03	1.94	0.80	1.14
35	1.78	3.49	0.93	1.85	0.64	1.00
45	1.78	3.49	0.92	1.85	0.64	1.00

**Table 2:** Result of angular resolution evaluation for the concave probe with a radius of 30 mm.

Depth. mm	Number of Elements in the Probe					
	16		32		64	
	Distance between Elements. mm					
	1	0.5	1	0.5	1	0.5
15	2.09	3.61	1.52	2.11	1.33	1.52
25	1.82	3.42	1.14	1.89	0.91	1.14
35	1.78	3.35	1.00	1.84	0.71	1.00
45	1.78	3.34	1.00	1.83	0.71	1.00

**Table 3:** Result of angular resolution evaluation for the convex probe with a radius of 60 mm.

Depth. mm	Number of Elements in the Probe					
	16		32		64	
	Distance between Elements. mm					
	1	0.5	1	0.5	1	0.5
15	1.90	3.61	1.33	2.09	1.18	1.33
25	1.84	3.53	1.06	1.94	0.87	1.14
35	1.80	3.49	1.00	1.85	0.74	1.00
45	1.81	3.49	0.99	1.85	0.74	1.00

**Table 4:** Result of angular resolution evaluation for the convex probe with a radius of 30 mm.

Depth. mm	Number of Elements in the Probe					
	16		32		64	
	Distance between Elements. mm					
	1	0.5	1	0.5	1	0.5
15	1.90	3.61	1.33	2.05	0.95	1.33
25	1.82	3.42	1.14	1.87	0.80	1.14
35	1.78	3.35	1.00	1.84	0.71	1.07
45	1.78	3.34	1.00	1.83	0.71	1.06

**Table 5:** Result of angular resolution evaluation for the convex probe with infinite curvature.

Depth. mm	Number of Elements in the Probe					
	16		32		64	
	Distance between Elements. mm					
	1	0.5	1	0.5	1	0.5
15	1.12	2.12	0.58	1.12	0.35	0.59
25	1.07	2.10	0.58	1.07	0.33	0.58
35	1.05	2.06	0.54	1.05	0.31	0.54
45	1.05	2,06	0.54	1.05	0.31	0.54

**Table 6:** Result of angular resolution evaluation using in situ experimental data.

**Distance of reflectors’ locations, mm**	15	20	25	35	45
**Angular resolution, °**	2.4	2.2	2.0	1.9	1.9

## Data Availability

The data that support the findings of this study are available from the corresponding author upon reasonable request.
